# Artificial targeting of misfolded cytosolic proteins to endoplasmic reticulum as a mechanism for clearance

**DOI:** 10.1038/srep12088

**Published:** 2015-07-14

**Authors:** Fen Liu, Deanna M. Koepp, Kylie J. Walters

**Affiliations:** 1 Protein Processing Section, Structural Biophysics Laboratory, Center for Cancer Research, National Cancer Institute, Frederick, MD 21702 USA; 2 Department of Genetics, Cell Biology and Development, University of Minnesota, Minneapolis, MN 55455 USA

## Abstract

We report that misfolded cytosolic proteins can be cleared from mammalian cells by directing them to endoplasmic reticulum (ER). NAT1 R64W and Parkin R42P are naturally occurring misfolded variants of cytosolic enzymes that acetylate arylamines and ubiquitinate proteins, respectively. We demonstrate that proteasome inhibition causes ER accumulation of NAT1 R64W and its ubiquitinated species, and that these products are cleared from cells following inhibition release. NAT1 WT by contrast is stable and not present at ER. The R42P mutation in Parkin locates to a UBL domain that interacts with C-terminal domains. Parkin R42P full length protein is trafficked poorly to ER and stable. Interestingly, fusion of the isolated R42P UBL to NAT1 WT results in a fusion product that is trafficked robustly to ER and degraded. Thus, the misfolded UBL is apparently masked by the intramolecular interactions. We also find that artificially directing Parkin R42P to ER by fusion with the Sec61β ER-directing transmembrane domain triggers its clearance. Altogether, our results suggest that routing misfolded cytosolic proteins to ER may be an effective strategy for clearance.

Structurally compromised or aggregated proteins are produced routinely in cells due to environmental stress, production errors, or inherited gene variations. Quality control mechanisms have been identified throughout the cell to counteract protein misfolding, as early as their synthesis at ribosomes^1^,^2^,^3^,^4^,^5^, and in multiple cellular compartments, including nucleus^6^ and ER (reviewed in 7). At ribosomes, there appears to be an active interplay between a complex network of molecular chaperones that facilitate nascent chain folding (reviewed in^8^) and co-translational ubiquitin-mediated degradation^1^,^2^,^4^,^5^, although the mechanism of ubiquitination is not yet well defined. Molecular chaperones in protein quality control pathways prevent aggregation and facilitate refolding of misfolded proteins^3^,^9^. Terminally misfolded proteins can be sequestered at specific cellular compartments^10^,^11^ and/or targeted for proteolysis by proteasome or lysosome.

Protein quality control frequently involves changes in sub-cellular localization. ERAD substrates are retrotranslocated to cytosol through a poorly defined mechanism for degradation by cytosolic proteasomes. Damaged mitochondria can be removed in bulk by autophagy-mediated lysosome turnover^12^; however, cytosolic proteasome also degrades inner mitochondrial membrane proteins^13^. In yeast, ER E3 ligase Doa10 is required for eliminating degron fused cytosolic proteins^*14,15*^, and in parallel or cooperation with Ubr1, nuclear E3 ligase San1 ubiquitinates a subset of engineered cytosolic misfolded proteins after chaperone-assisted import into nuclei^*16*^. A nuclear pathway to degrade misfolded cytosolic proteins may also exist in mammalian cells and similarly require molecular chaperones for nuclear import^18^. A plethora of ligases and sub-cellular locations is invoked to ubiquitinate mutant superoxide dismutase-1 (SOD1) in mammalian cells. It is ubiquitinated at ER by gp78^19^, mitochondria by MITOL/MARCH5^20^ and by cytosolic ligase RNF19A/Dorfin^21^. It is not yet known whether these events occur independently.

Inefficient clearance of misfolded proteins is associated with dysfunction and disease, including cancer and neurodegeneration. To date, quality control pathways for misfolded cytosolic proteins in mammalian cells are only vaguely understood, and it is not known how certain disease-associated misfolded proteins evade such systems. We used human arylamine N-acetyltransferase 1 (NAT1) and its R64W variant, as well as Parkin and its R42P variant, to study cytosolic protein quality control in mammalian cells. NAT1 R64W and Parkin R42P are products of naturally occurring DNA polymorphisms that associate with cancer and Juvenile Parkinsonism, respectively. NAT1 R64W retains enzymatic activity *in vitro*^22^, but aggregates^22^ and *in vivo*, exhibits reduced cellular abundance and activity^23^ due to constitutive degradation^24^. NAT1 polymorphisms are associated with cancer^*25,26*^, probably as a result of their constitutive removal from cells. NAT1 “wild-type” (WT) does not contain the R64W substitution and exhibits neither loss of enzymatic activity^23^, nor constitutive ubiquitination in human cells^24^. By comparing the behavior of NAT1 WT with NAT1 R64W, we present evidence that this structurally compromised protein is directed to ER for degradation by proteasome. The Parkin R42P substitution is situated in an N-terminal UBL domain, and when this region is isolated from the rest of the protein, causes unfolding^27^. We find that full length Parkin with the R42P mutation incorporated is trafficked poorly to ER and fails to be cleared. We are able to trigger Parkin R42P clearance from cells, however, by fusing it to the Sec61β C-terminal transmembrane domain that signals for ER localization. We also present evidence that Parkin intramolecular interactions involving the R42P UBL domain mask the presence of this structurally compromised domain and its ability to serve as a signal for degradation. Altogether, our work suggests that routing misfolded proteins to ER may be an effective strategy for their clearance. This finding has implications for the clearance of protein variants with gain-of-function or dysregulated activity, such as oncogenes introduced by microbial invaders.

## Results

### Naturally occurring NAT1 R64W co-localizes with ER marker Sec61β in MG132-treated COS7 cells

**Figure 1:**
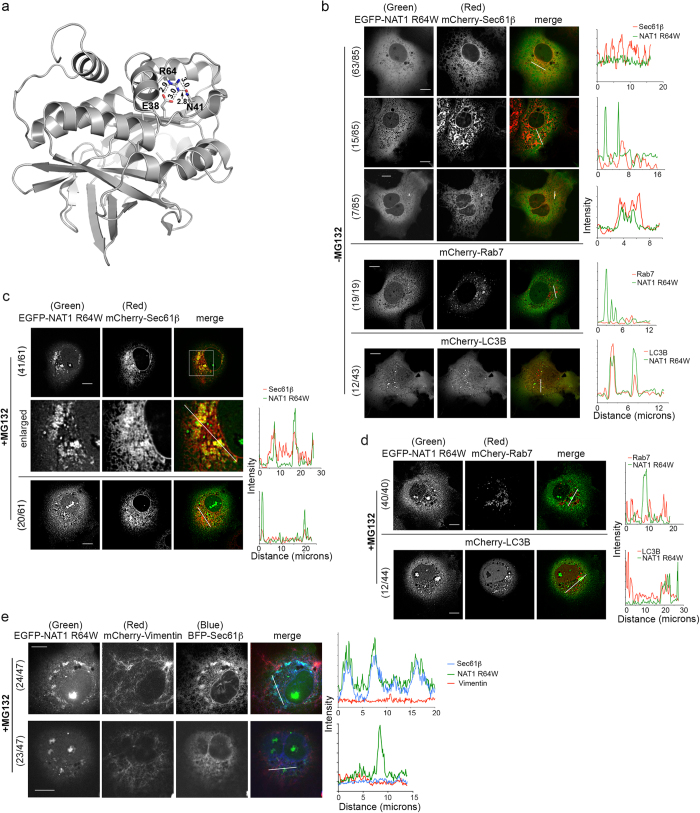
The naturally occurring R64W substitution in NAT1 causes it to be routed to ER. (**a**) Ribbon diagram of human NAT1 (PDB: 2PQT) displaying R64 contacts with E38 and N41. (**b**–**d**) COS7 cells co-expressing EGFP-NAT1 R64W and mCherry-Sec61β, mCherry-Rab7, or mCherry-LC3B were visualized by spinning disk live cell confocal microscopy without (**b**) or with (**c**,**d**) MG132 treatment. (**e**) MG132-treated COS7 cells co-expressing EGFP-NAT1 R64W, mCherry-Vimentin and BFP-Sec61β were visualized by live cell confocal microscopy. Intensity profiles are displayed for the region indicated by a white line in the merged image ((**b**–**e**), right), and a 10 μm scale bar is included in the left panels of (**b**–**e**). Fractions to the left of the images in (**b**–**e**) indicate the number of cells showing the representative phenotype over the total number of cells examined.

NAT1 R64 forms electrostatic interactions with E38 and N41 (Fig. 1a), and its replacement with tryptophan causes protein aggregation^22^. To investigate whether this arginine to tryptophan substitution alters the protein localization in cells, EGFP-NAT1 R64W or WT was transiently expressed in COS7 cells with mCherry-Sec61β (ER marker), -Rab7 (late endosomal marker) or -LC3B (autophagy marker). Live cell spinning disc confocal microscopy revealed NAT1 WT diffuse throughout the cytosol and nucleus without (Supplementary Fig. 1a) or with (Supplementary Fig. 1b, top) 4-hour MG132 treatment. In 32% (27/84 examined) of MG132-treated cells, NAT1 WT formed small puncta (Supplementary Fig. 1b). No co-localization was observed for NAT1 WT with Sec61β (Supplementary Fig. 1a, top panel, 1b, top two panels), Rab7 (Supplementary Fig. 1a, middle panel, 1b, third panel) or LC3B (Supplementary Fig. 1a, 1b, bottom), regardless of cell treatment or the presence of puncta.

NAT1 R64W was similarly diffuse throughout the cytosol in the absence of MG132 (Fig. 1b, top panel) and, in 26% (22/85 examined) of cells localized to bright, punctuated structures (Fig. 1b). The majority of NAT1 R64W puncta did not co-localize with ER marker Sec61β (Fig. 1b, second panel); however, 8% (7/85 examined) of cells contained puncta that co-localized with Sec61β (Fig. 1b, third panel). No cell exhibited co-localization between NAT1 R64W and endosomal marker Rab7, with 19 cells closely scrutinized (Fig. 1b, fourth panel). Partial co-localization was observed in 28% (12/43 examined) of untreated cells between NAT1 R64W puncta and autophagy marker LC3B (Fig. 1b, bottom panel).

After 4-hour MG132 treatment, 67% (41/61 examined) of cells demonstrated NAT1 R64W to accumulate in structures that contain Sec61β (Fig. 1c, top two panels). This finding suggests NAT1 R64W presence at ER. No co-localization was observed between NAT1 R64W and Rab7 under these conditions with 40 cells examined closely (Fig. 1d, top panel). Partial co-localization was observed in 27% (12/44 examined) of cells between autophagy marker LC3B and NAT1 R64W (Fig. 1d, bottom panel). Thus, a population of NAT1 R64W was identified at ER in MG132-treated COS7 cells, and at autophagic vesicles.

Aggresomes are cellular deposits of aggregated and ubiquitinated proteins that contain Vimentin. In MG132-treated COS7 cells expressing mCherry-Vimentin, EGFP-NAT1 R64W and BFP-Sec61β, 51% of examined cells (24/47 cells) demonstrated NAT1 R64W co-localized with Sec61β, and not Vimentin (Fig. 1e, top). NAT1 R64W therefore appears to generally accumulate at ER in COS7 cells, and not at aggresomes, following proteasome inhibition.

### Ubiquitinated NAT1 R64W accumulates at rough ER in MG132-treated 293T cells

**Figure 2:**
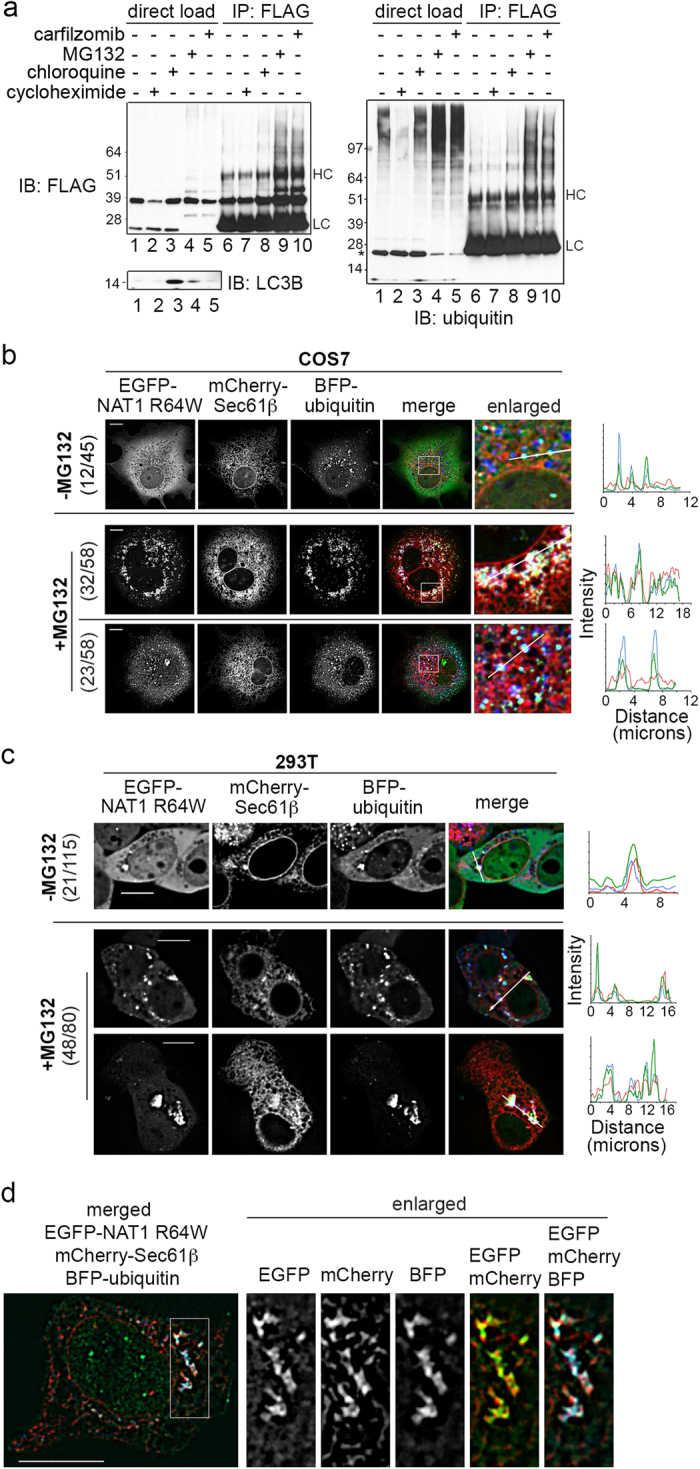
NAT1 R64W accumulates at structures containing both ER marker Sec61β and ubiquitin upon proteasome inhibition. (**a**) 293T cells expressing FLAG-NAT1 R64W were treated with carfilzomib, MG132, chloroquine or cycloheximide and lysed in 7.2 M urea-supplemented RIPA buffer. Lysates were subjected to immunoprecipitation with anti-FLAG antibody and immunoprobed with anti-ubiquitin (right) and anti-FLAG (left, top) antibodies. Direct loads are included with additional immunoprobing with anti-LC3B antibody (left, bottom). HC, heavy chain; LC, light chain. *indicates a non-specific band from the ubiquitin antibody. (**b**,**c**) COS7 (**b**) or 293T (**c**) cells expressing EGFP-NAT1 R64W, mCherry-Sec61β and BFP-ubiquitin were visualized by live cell microscopy with (bottom) or without (top) MG132 treatment. MG132-treated cells with varying co-localization are displayed. Intensity profiles are displayed for the regions indicated with white bars. Fractions to the left of the images indicate the number of cells showing the representative phenotype over the total number of cells examined. (**d**) MG132-treated 293T cells transfected as in (**c**) were subjected to methanol fixation and imaged by super-resolution SIM. A 10 μm scale bar is included in the left panels of (**b**–**d**).

Proteasome and lysosome are the major degradation machineries in eukaryotes to clear misfolded proteins. Various inhibitors were used to test the contribution of these two machineries in NAT1 R64W clearance. 293T cells expressing FLAG-tagged NAT1 R64W were treated for six hours with proteasome inhibitors MG132 or carfilzomib, or lysosome inhibitor chloroquine. Following lysis in 7.2 M urea supplemented RIPA buffer, the status of NAT1 R64W was assessed by immunoprecipitation with anti-FLAG antibody and immunoprobing with anti-FLAG or anti-ubiquitin antibodies. NAT1 R64W exhibited reduced protein levels after 6-hour cycloheximide treatment to inhibit protein synthesis (Fig. 2a, left panel, lane 2); this finding is consistent with its constitutive degradation^22^,^24^. Higher molecular weight species characteristic of ubiquitinated NAT1 R64W were significantly enhanced with MG132 or carfilzomib (Fig. 2a, left panel, lanes 4, 5 and 9, 10), but not with chloroquine (Fig. 2a, left panel, lane 3 and 8). Immunodetection with anti-ubiquitin antibody confirmed the accumulation of ubiquitinated NAT1 R64W by proteasome inhibitors, but not lysosome inhibitor (Fig. 2a, right panel, lane 8–10). As a control, we immunoprobed the cell lysates for lysosomal substrate LC3B-II and found it to accumulate as expected with chloroquine treatment (Fig. 2a, bottom panel, lane 3). These data suggests that the ubiquitin-proteasome pathway is the major degradation machinery to dispose of NAT1 R64W.

To test whether NAT1 R64W that accumulates at perinuclear ER upon proteasome inhibition (Fig. 1c) contains ubiquitinated species, live cell imaging was performed with COS7 or 293T cells expressing EGFP-NAT1 R64W/WT, mCherry-Sec61β (ER marker) and BFP-ubiquitin. In both cell lines, NAT1 WT was diffusely distributed throughout the cytosol and did not co-localize with Sec61β or ubiquitin without MG132 treatment in all cells examined (Supplementary Fig. 2a,b, top). When COS7 cells were treated with MG132, no co-localization was observed for NAT1 WT with either ubiquitin or Sec61β; however, 53% of examined cells (34/64 cells) demonstrated Sec61β and ubiquitin to co-localize (Supplementary Fig. 2a, bottom). Such co-localization was reduced to 21% (10/48 examined cells) in MG132-treated 293T cells (Supplementary Fig. 2b, middle) and 13% (6/48 cells) exhibited small puncta containing NAT1 WT co-localized with ubiquitin and not Sec61β (Supplementary Fig. 2b, bottom).

In 27% (12/45 examined) of untreated COS7 cells, NAT1 R64W formed puncta that co-localized with ubiquitin, but not Sec61β (Fig. 2b, top, light blue), whereas NAT1 R64W, ubiquitin and Sec61β were all co-localized in 9% (4/45 cells). Similarly, 18% (21/115 cells) of untreated 293T cells exhibited such puncta, with all three proteins co-localized (Fig. 2c, top, white). MG132 treatment caused NAT1 R64W to accumulate and co-localize to varying degrees with both Sec61β and ubiquitin in 55% (32/58) of COS7 cells (Fig. 2b, second panel), with 40% (23/58 cells) showing NAT1 R64W co-localization with ubiquitin but not Sec61β (Fig. 2b, bottom); no co-localization between NAT1 R64W with either ubiquitin or Sec61β was observed for 5% (3/58) of cells. 60% (48/80 examined) of MG132-treated 293T cells exhibited NAT1 R64W accumulated at structures that contained both ubiquitin and Sec61β (Fig. 2c, bottom two panels). MG132-treated 293T cells were also fixed and imaged by super-resolution structured illumination microscope (SIM), which confirmed co-localization of EGFP-NAT1 R64W, mCherry-Sec61β and BFP-ubiquitin (Fig. 2d).

**Figure 3:**
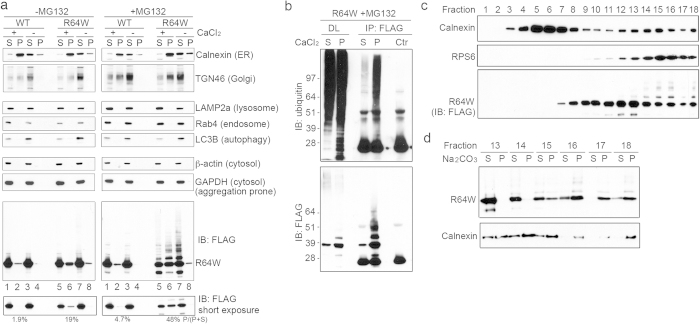
NAT1 R64W and its ubiquitinated species are present at rough ER. (**a**) 293T cells were transfected with FLAG-NAT1 WT or FLAG-NAT1 R64W and lysed by homogenization. CaCl_2_ was added (+) or not (as a control, −) to PMFs to generate an ER-enriched pellet (P) and supernatant (S) without (left) or with (right) MG132 treatment. Immunoblotting was used to probe for marker proteins of various cellular components, as indicated. The bottom immunoblot was quantified by ImageJ, with the indicated percentage representing band intensity in the corresponding pelleted fraction divided by the sum of that pellet and the associated supernatant. (**b**) The pellet (P) and supernatant (S) fractions after CaCl_2_ precipitation from MG132-treated NAT1 R64W expressing 293T cells were subjected to immunoprecipitation with anti-FLAG antibody and immunoprobed with anti-ubiquitin or anti-FLAG antibodies. The control immunoprecipitation (Ctr) was done with non-transfected 293T cells. DL, direct load. (**c**) The PMF from MG132-treated, NAT1 R64W expressing 293T cells was subjected to sucrose gradient fractionation and immunoprobed with indicated antibodies. (**d**) Sodium carbonate buffer was added to fractions 13–18 from (**c**) to extract peripheral and luminal proteins from the membrane. Supernatant (S) and pelleted (P) fractions after extraction were immunoprobed with antibodies indicated.

To test further the presence of ubiquitinated NAT1 R64W at ER, a CaCl_2_ precipitation protocol was used for ER enrichment^28^. This protocol features the use of low centrifuge speed to enrich for ER membranes, avoiding co-sedimentation of aggregates, as observed by conventional ultracentrifugation. Contribution from aggregation was readily scrutinized by applying the identical procedure without CaCl_2_ addition. In brief, CaCl_2_ was added to the post-mitochondrial fraction (PMF) from NAT1 R64W or WT expressing 293T cells to generate an ER-enriched pellet after centrifugation at 8,000 g. Immunoprobing revealed the presence of ER, as assessed by Calnexin (Fig. 3a, left and right panels, lanes 2, 6), with some contamination from the trans-Golgi network (TGN46) (Fig. 3a, left and right panels, lanes 2, 6). This fraction contained no apparent marker for lysosome (LAMP2a), endosome (Rab4), autophagic vesicles (LC3B), or cytosolic proteins (β-actin, GAPDH) (Fig. 3a, left and right panels, lanes 2, 6). 19% of detected NAT1 R64W was observed in the ER-enriched fraction, where only 1.9% NAT1 WT was detected (Fig. 3a, left panel, bottom two, compare lane 2 with lane 6). 6-hour MG132 treatment does not significantly enhance the presence of NAT1 WT (4.7% detected) in the ER-enriched fraction (Fig. 3a, right panel, bottom, lane 2); however, higher molecular weight species of NAT1 R64W and 48% of unmodified NAT1 R64W accumulated in this fraction following MG132 treatment (Fig. 3a, right panel, bottom two panels, lane 6).

The supernatant and pelleted fractions from MG132-treated, NAT1 R64W expressing 293T cells were used to immunoprecipitate NAT1 with anti-FLAG antibody under denaturing condition and ubiquitin immunoprobed. This experiment confirmed the presence of ubiquitinated NAT1 R64W in the pelleted fraction (Fig. 3b). The precipitation of NAT1 R64W into this ER-enriched fraction is not due to aggregation, since PMF subjected to the identical procedure without CaCl_2_ revealed only a slight presence of NAT1 R64W (Fig. 3a, left and right panels, bottom, lane 8). GAPDH, a cytosolic aggregation-prone protein^29^ was not pelleted by CaCl_2_ (Fig. 3a, left and right panels, lanes 2 and 6).

To further interrogate the ER association of NAT1 R64W and its ubiquitinated species, we performed sucrose gradient fractionation^30^ on the PMF of MG132-treated 293T cells expressing NAT1 R64W. The ER membrane protein Calnexin peaked at two positions (Fig. 3c, top), spanning fractions 4–8 and 13–16. Consistent with the literature^30^, the heavier species (13–16) demonstrated the presence of ribosomal marker RPS6, thus assigning these fractions to rough ER microsomes (Fig. 3c, second panel). A portion of NAT1 R64W, and especially its high molecular weight species, fractionated into these rough ER microsome fractions (Fig. 3c, third panel). The earlier ER fractions at 4–8 are most likely smooth ER, and did not demonstrate a presence for NAT1 R64W. This result is consistent with the CaCl_2_ precipitation experiment (Fig. 3a), which precipitates rough ER^28^.

To determine whether NAT1 R64W at ER associates with membranes, we performed a Na_2_CO_3_ extraction experiment for fractions 13–18. This experiment revealed greater NAT1 R64W retained in the pellet for heavier fractions following Na_2_CO_3_ extraction (Fig. 3d). Na_2_CO_3_ extracts peripheral and luminal proteins from vesicles, leaving membrane proteins and those strongly associated with membranes in the pellet. Thus, NAT1 R64W appears to accumulate and tightly associate with rough ER after MG132 treatment.

### ER localized NAT1 R64W is cleared following relief from proteasome inhibition

**Figure 4:**
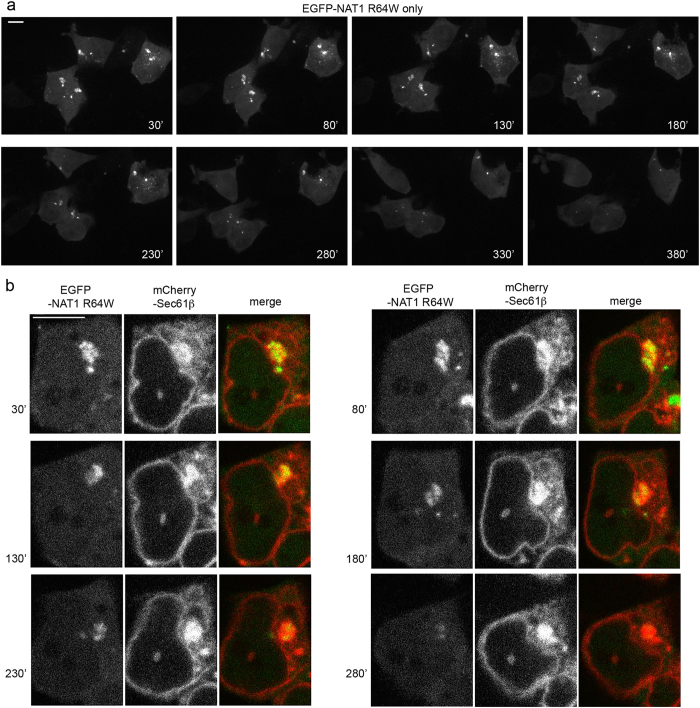
ER localized NAT1 R64W is cleared following relief from proteasome inhibition. (**a**,**b**) 293T cells expressing EGFP-NAT1 R64W and mCherry-Sec61β were treated with MG132 for 6 hours and then exchanged to fresh media containing cycloheximide and lacking MG132. The cells were imaged every 5 minutes by a spinning disc confocal microscope. Maximum intensity projections for EGFP (**a**) and one representative optical section for EGFP and mCherry (**b**) are displayed at the indicated time following exchange. A 10 μm scale bar is included in the first panel of (**a**) and (**b**), respectively.

To test whether ER-accumulated NAT1 R64W can be degraded, we monitored MG132-treated 293T cells expressing EGFP-NAT1 R64W and mCherry-Sec61β every five minutes after exchange to MG132-free media containing cycloheximide. NAT1 R64W disappeared over time (Fig. 4a, see also Supplementary Movie 1) and in some cases, co-migrated with mCherry-Sec61β until clearance (Fig. 4b, see also Supplementary Movie 2). The disappearance of NAT1 R64W was not from photobleaching, as EGFP signal is retained in cells imaged identically, but without cycloheximide (Supplementary Fig. 3). Without cycloheximide, NAT1 R64W continues to accumulate and some cells die (Supplementary Fig. 3 and Movie 3).

Altogether, these results suggest that NAT1 R64W is degraded by proteasome following an ER-directed route; however, we expect that this pathway to clearance is not the only mechanism available to remove this misfolded protein. We also observed the clearance of NAT1 R64W puncta that did not appear to co-localize with Sec61β (Supplementary Movie 2), and as noted above, partial co-localization is observed with autophagy marker LC3B (Fig. 1b,d, bottom panels).

### Parkin R42P is poorly trafficked to ER and stable in mammalian cells

**Figure 5:**
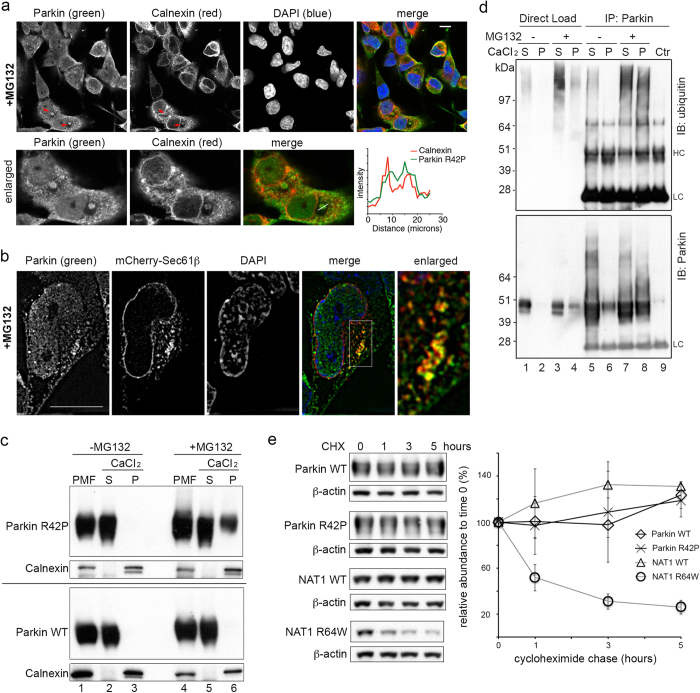
Parkin R42P is poorly trafficked to ER and stable in mammalian cells. (**a**) Neuroblastoma SHSY5Y cells stably expressing Parkin R42P were treated with MG132 for 7 hours and immunofluorescence was done with mouse anti-Parkin (green) and rabbit anti-Calnexin (red) antibodies. Arrows indicate structures that contain both Parkin and Calnexin and cells containing such structures were enlarged for better viewing (bottom). A scale bar representing 10 μm is included in the upper right panel. (**b**) Neuroblastoma SHSY5Y cells stably expressing Parkin R42P were transfected with mCherry-Sec61β and treated with MG132 for 7 hours. Cells were fixed and Parkin immunostained (green) with mouse anti-Parkin antibody and subjected to SIM. A scale bar represents 10 μm. (**c**) Parkin R42P (top) or WT (bottom) expressing SHSY5Y cells were treated with (right) or without (left) MG132 for 7 hours and CaCl_2_ was added to the PMF to enrich for ER in a gently pelleted fraction (P), as illustrated in Fig. 3a. Immunodetection was achieved with anti-Parkin and anti-Calnexin antibodies. (**d**) An ER-enriched pellet (P) and soluble (S) fractions from Parkin R42P expressing SHSY5Y cells treated with (+) or without (−) proteasome inhibitor MG132 for 7 hours were generated as in (**c**). Samples were immunoprecipitated with anti-Parkin antibody (IP) and immunoprobed with anti-ubiquitin antibody (top) or anti-Parkin antibody (bottom). Control immunoprecipitation (Ctr) was done with cells not expressing Parkin R42P. (**e**) SHSY5Y cells expressing Parkin WT or Parkin R42P and 293T cells expressing NAT1 WT or NAT1 R64W were treated with cycloheximide and cells harvested at 0, 1, 3, 5 hours post-treatment. Cells were lysed by 1% Triton buffer and supernatants subjected to immunoprobing, as indicated (left). Results from three independent experiments were quantified by ImageJ normalizing to β-actin, and the mean and standard deviation plotted (right).

The R42P substitution in Parkin is reported to render its isolated N-terminal UBL domain unfolded^27^ and we therefore tested whether Parkin R42P is also directed to ER. Parkin R42P stably expressed in an SHSY5Y neuroblastoma cell line^31^ was diffuse throughout the cytosol with bright tiny puncta in some cells, but did not exhibit co-localization with ER marker Calnexin (Supplementary Fig. 4a). When treated with MG132 for 7 hours, 17% of (15/87 examined) cells indicated Parkin R42P to localize at structures that also contain Calnexin (Fig. 5a, red arrows). To better investigate co-localization between Parkin R42P and ER, we expressed mCherry-Sec61β in this Parkin R42P expressing SHSY5Y cell line and treated the cells for 7 hours with MG132. The cells were fixed and imaged by SIM to reveal partial co-localization for Parkin R42P and Sec61β (Fig. 5b). The relative abundance of Parkin R42P at ER however was less compared to NAT1 R64W (Fig. 2c,d).

We tested further whether Parkin R42P localizes to ER by using CaCl_2_ and low speed centrifugation to enrich for ER, as described above for NAT1 (Fig. 3a). Neither Parkin WT nor Parkin R42P was detected in the ER-enriched fraction without MG132 treatment (Fig. 5c, lane 3). The presence of Parkin R42P (top), and not Parkin WT (bottom), was detected in this fraction from cells treated for 7 hours with MG132 (Fig. 5c, lane 6). The relative abundance of Parkin R42P in this fraction however was significantly reduced compared to NAT1 R64W (Fig. 3a). Higher molecular weight Parkin R42P species characteristic of protein ubiquitination were also less observable with direct immunoblotting (Fig. 5c) compared to NAT1 R64W (Fig. 3a).

We tested for the presence of ubiquitinated Parkin R42P in CaCl_2_ precipitated pelleted and supernatant fractions from untreated cells and those treated with MG132 for 7 hours. Parkin was immunoprecipitated in denaturing conditions with anti-Parkin antibody and ubiquitin probed by immunoblotting with anti-ubiquitin antibody. MG132 enhanced the presence of ubiquitinated Parkin R42P, which was observed in both the pelleted and supernatant fractions after MG132 treatment (Fig. 5d, lanes 7 and 8). These experiments indicate a presence for Parkin R42P and its ubiquitinated species at ER in SHSY5Y cells upon proteasome inhibition, but at a reduced level compared to NAT1 R64W.

We used a cycloheximide chase experiment to assay protein stability for NAT1 WT, NAT1 R64W, Parkin WT, and Parkin R42P. We found that Parkin R42P was stable after five hours of cycloheximide treatment, with similar stability compared to Parkin WT and NAT1 WT. By contrast, NAT1 R64W was degraded with a half-life of less than two hours (Fig. 5e). To test whether the disparate degradation rate of Parkin R42P and NAT1 R64W originates from using different cell types (SHSY5Y cells for Parkin and 293T cells for NAT1), we expressed myc-Parkin R42P in 293T cells and FLAG-NAT1 R64W in SHSY5Y cells. We similarly observed NAT1 R64W to be readily degraded and Parkin R42P stability (Supplementary Fig. 4b). Altogether, our data indicate that NAT1 R64W is robustly trafficked to ER and cleared from cells, whereas Parkin R42P is poorly trafficked to ER and stable.

### NAT1 WT is directed to ER and cleared from cells when fused to Parkin R42P UBL domain

**Figure 6:**
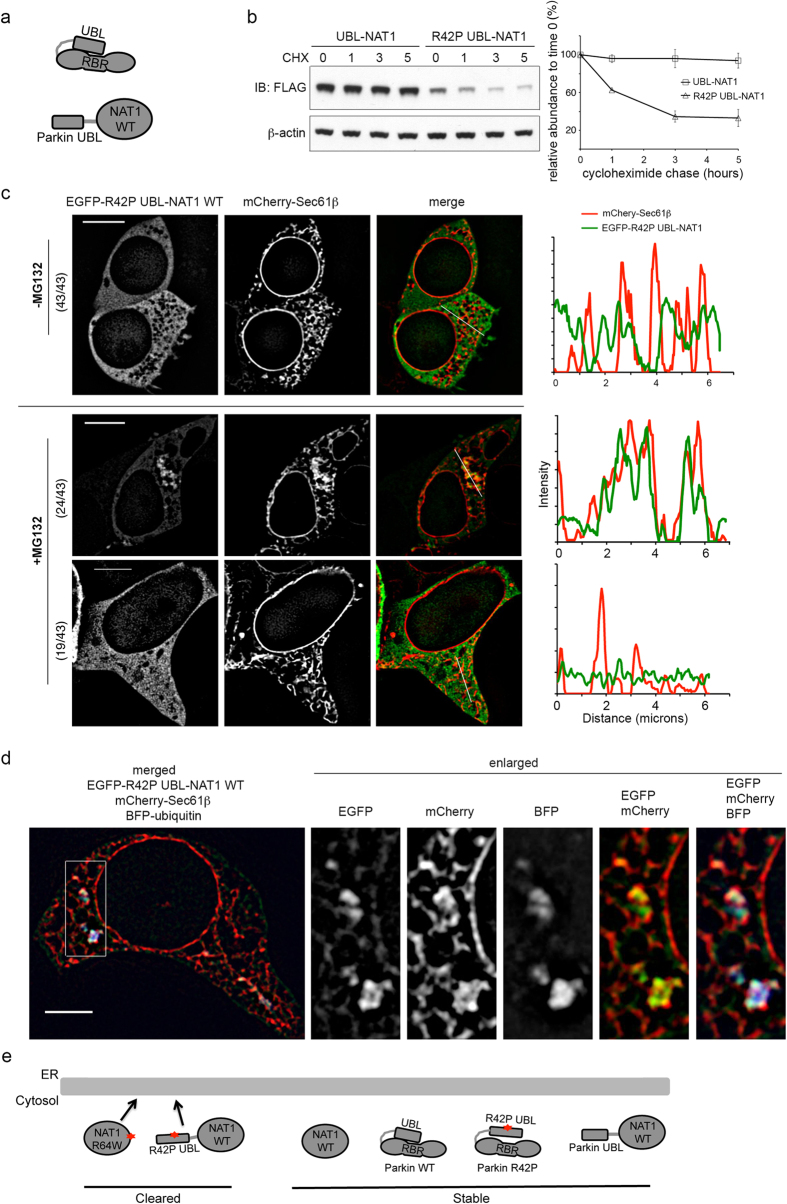
Parkin R42P UBL domain triggers ER localization and degradation of NAT1 WT when fused to its N-terminal end. (**a**) Schematic representation of Parkin intramolecular interaction between the N-terminal UBL domain and the RBR (top) and depiction of a construct with Parkin UBL domain fused to the N-terminal end of NAT1 WT (bottom). (**b**) 293T cells expressing Parkin UBL-NAT1 WT or Parkin R42P UBL-NAT1 WT were treated with cycloheximide and harvested at 0, 1, 3, 5 hours post-treatment. Cells were lysed by 1% Triton buffer and supernatants subjected to immunodetection with anti-FLAG (top) or anti-actin (bottom) antibodies (left). Results from three independent experiments were quantified by ImageJ normalizing to β-actin, and the mean and standard deviation plotted (right). (**c**) 293T cells expressing EGFP-Parkin R42P UBL-NAT1 and mCherry-Sec61β without (top) and with (bottom) MG132 treatment were subjected to live cell imaging. Intensity profiles are displayed for regions highlighted with a white bar. Fractions to the left of the images indicate number of cells showing the representative phenotype over the total number of cells examined. (**d**) MG132-treated 293T cells expressing EGFP-Parkin R42P UBL-NAT1, BFP-ubiquitin and mCherry-Sec61β were subjected to methanol fixation and imaged with super-resolution SIM. A 5 μm scale bar is included in the left panel of (**c**) and (**d**). (**e**) Schematic summary depicting ER localization and clearance for NAT1 R64W and Parkin R42P UBL-NAT1 WT contrasted to the stability of NAT1 WT, Parkin WT, Parkin R42P, and Parkin WT UBL-NAT1 WT; these stable proteins are not targeted to ER.

The Parkin UBL and RBR (RING-In Between RING-RING) domains interact intramolecularly (Fig. 6a, top) to inhibit autoubiquitination of this E3 ligase^32^. We reasoned that residual interaction between Parkin R42P UBL and the RBR domain may play a role in the failure to triage Parkin R42P, as autoubiquitination is reduced for this variant compared to other Parkin variants with UBL domain mutations as shown in 32. Alternatively, the presence of structured C-terminal domains may also disengage cellular surveillance systems for misfolded proteins. NAT1 WT is stable (Fig. 5e) and does not localize to ER in mammalian cells (Supplementary Fig. 1a,b). To test whether the C-terminal part of Parkin R42P contributes to its stability, we fused Parkin WT or R42P UBL domain to the N-terminal end of NAT1 WT (Fig. 6a, bottom). Whereas Parkin UBL-NAT1 was stable (Fig. 6b), Parkin R42P UBL-NAT1 was cleared from cells (Fig. 6b).

We used microscopy techniques to test whether Parkin R42P UBL-NAT1 co-localizes with Sec61β in untreated 293T cells and following MG132 treatment for 6 hours. In untreated cells, live cell confocal microscopy demonstrated Parkin R42P UBL-NAT1 WT to be diffuse throughout the cytosol without appreciable co-localization with Sec61β (Fig. 6c, top panel) in all 43 cells examined. Co-localization of Parkin R42P UBL-NAT1 was readily observed in 56% (24/43 examined) of MG132-treated 293T cells by confocal live cell imaging (Fig. 6c, middle panel). Super-resolution SIM after fixation validated this finding, demonstrating co-localization between Parkin R42P UBL-NAT1 and Sec61β as well as ubiquitin (Fig. 6d). These results indicate that loss of the region C-terminal to the UBL domain enables Parkin R42P UBL to serve as a signal for ER recruitment and protein clearance. Altogether, our data correlate localization of misfolded cytosolic proteins to ER with their clearance, such that efficient ER targeting leads to robust degradation (Fig. 6e).

### Artificial tagging of full length Parkin to ER leads to its clearance from cells

**Figure 7:**
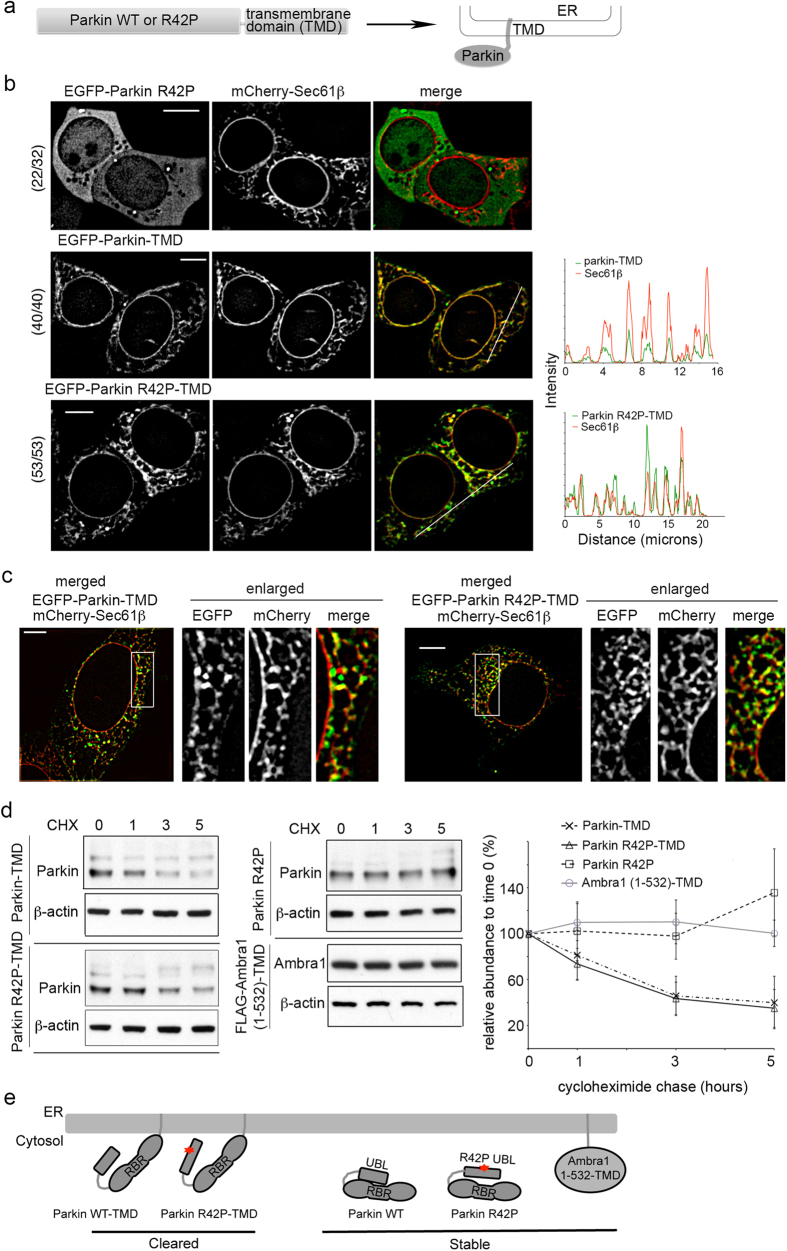
Fusion of an ER localization signal onto Parkin causes it to be cleared from cells. (**a**) Schematic representation of a construct with Parkin WT or Parkin R42P fused at the C-terminal end to the Sec61β transmembrane domain. (**b**) 293T cells expressing EGFP-Parkin, EGFP-Parkin WT-TMD or EGFP-Parkin R42P-TMD and mCherry-Sec61β were subjected to live cell imaging. Intensity profiles are displayed for the regions highlighted with a white bar. Fractions to the left of the images indicate the number of cells showing the representative phenotype over the total number of cells examined. (**c**) 293T cells treated as in (**b**) were subjected to methanol fixation and imaged with super-resolution SIM. A 5 μm scale bar is included in the left panels of (**b**) and (**c**). (**d**) 293T cells expressing Parkin R42P, Parkin WT-TMD, Parkin R42P-TMD, or FLAG-Ambra1 (1–532)-TMD were treated with cycloheximide and harvested at 0, 1, 3, 5 hours post-treatment. Cells were lysed by 1% Triton buffer and supernatants subjected to immunoblotting with anti-Parkin or anti-actin antibodies (left and middle). Results from three independent experiments were quantified by ImageJ normalizing to β-actin, and the mean and standard deviation plotted (right). (**e**) Schematic representation illustrating ER tethering and resulting degradation of Parkin WT and R42P by the Sec61β transmembrane domain (TMD). Ambra1 (1–532)-TMD is not cleared from cells and we propose that ER tethering may interfere with Parkin UBL:RBR intramolecular interactions, thus disrupting Parkin WT structural integrity.

We next tested whether tethering full length Parkin R42P or WT directly to ER triggers its clearance. The C-terminal transmembrane domain (TMD) from tail-anchored ER membrane protein Sec61β was fused to the C-terminus of Parkin R42P or Parkin WT in attempt to direct the protein products to ER (Fig. 7a). EGFP-Parkin R42P was largely diffuse throughout the cytosol in all 32 cells examined without appreciable co-localization with Sec61β and with 69% of cells exhibiting puncta (Fig. 7b, top). As expected, EGFP-Parkin R42P-TMD (all 53 cells analyzed) and EGFP-Parkin-TMD (all 40 cells analyzed) exhibited a reticular distribution that co-localized with ER marker Sec61β as revealed by confocal live cell imaging (Fig. 7b). This co-localization was confirmed by super-resolution SIM (Fig. 7c); these experiments were performed without MG132 treatment.

To test for protein stability, cycloheximide chase experiments were performed with Parkin R42P, Parkin R42P-TMD and Parkin WT-TMD. Parkin R42P remained stable after 5 hours of cycloheximide chase (Fig. 7d, middle panel, top). By contrast, Parkin R42P-TMD (Fig. 7d, left panel, bottom) and Parkin WT-TMD (Fig. 7d, left panel, top) were degraded with a half-life of less than three hours (Fig. 7d, right panel). We were surprised that Parkin WT-TMD was degraded when localized to ER. We therefore used Ambra1, which binds Parkin and functions in Parkin-mediated mitophagy^33^, to test whether ER tethering of cytosolic proteins generally triggers their clearance. We found that Ambra1 (1–532)-TMD was stable over five hours (Fig. 7d, middle panel, bottom), indicating that recruitment to ER is not sufficient to trigger protein degradation. This finding suggests selectivity at the ER, and that perhaps C-terminal TMD fusion to Parkin disrupts its structural integrity (Fig. 7e).

## Discussion

The results in this manuscript tightly couple robust ER localization of misfolded cytosolic proteins to their clearance. NAT1 R64W is a naturally occurring variant that is constitutively cleared via the ubiquitin-proteasome system. Although NAT1 WT protein is not associated with ER (Supplementary Fig. 1), NAT1 R64W is robustly trafficked there (Fig. 1c). This variant and its ubiquitinated species accumulate at ER when proteasome is inhibited (Fig. 2), but also associates with ER when degradation is not compromised. In untreated 293T cells, NAT1 R64W was present in an ER-enriched fraction (Fig. 3a, left panel), and in 18% of these cells, formed puncta that co-localize with mCherry-Sec61β and BFP-ubiquitin (Fig. 2c, top). NAT1 R64W that accumulates at ER upon proteasome inhibition is readily cleared following relief from inhibition (Fig. 4). These findings are consistent with a model in which degradation by proteasome is facilitated for this misfolded cytosolic proteins by routing to ER.

ERAD is well characterized from studies in yeast, with distinct ubiquitination machinery at the ER to triage secretory and membrane proteins that naturally passage through the ER, as reviewed in^34^. The degradation pathway used by NAT1 R64W is unique from ERAD in that this protein does not traffic through ER in its healthy, properly folded state. The molecular constituents that trigger ER trafficking of misfolded NAT1 R64W remain to be identified; however, it is noteworthy that we observed NAT1 R64W fractionation with ribosome-associated ER rather than smooth ER (Fig. 3c). This finding may suggest that NAT1 R64W is identified by cellular surveillance systems during or directly following protein synthesis. Furthermore, we speculate that localization of NAT1 R64W at the ER ultimately leads to its degradation by proteasome; for example, we observe ER-accumulated NAT1 R64W to be cleared when proteasome inhibition is relieved (Fig. 4).

We hypothesized that Parkin R42P evades quality control mechanisms because its misfolded UBL domain is masked by intramolecular interaction with structured C-terminal domains^32^. In support of this model, Parkin R42P UBL domain fusion to NAT1 WT led to ER localization (Fig. 6c) and protein clearance (Fig. 6b), whereas these phenomena were not observed with wild-type Parkin UBL domain.

Direct targeting to ER by fusion with the Sec61β transmembrane domain (Fig. 7a) caused Parkin R42P and Parkin WT to be cleared (Fig. 7d). Future studies are needed to investigate why Parkin WT-TMD was cleared at the ER; however, it is possible that docking against the ER membrane disrupts structural integrity of the C-terminal RBR domain or its interaction with the N-terminal UBL. Ambra1 (1–532)-TMD remained stable in cells (Fig. 7d, middle panel, bottom), demonstrating that recruitment to ER is not sufficient for triggering protein clearance. This result is expected as a sophisticated quality control system is in place at ER for resident and passenger proteins, as reviewed in 34.

In conclusion, we report an ER-directed route that facilitates the proteasomal degradation of misfolded cytosolic proteins. While only a subset of proteins may go through this pathway efficiently, as exemplified by NAT1 R64W versus Parkin R42P, artificial tagging to ER appears to be an effective manner for triggering clearance mechanisms. Future experiments are needed to identify the molecular determinants for NAT1 R64W versus Parkin R42P trafficking to ER, which may inspire avenues for the design of molecules that promote the clearance of pathogenic proteins.

## Methods

### Mammalian cell culture conditions

SHSY5Y cells expressing Parkin WT / R42P (from Dr. Poul H. Jensen, University of Aarhus, Denmark), COS7 and 293T cells (ATCC) were grown at 37 °C in DMEM supplemented with 10% FBS in a humidified atmosphere of 5% CO_2_ (25 μg/mL Zeocin was present in the media of SHSY5Y cells to maintain selection). Plasmid DNAs were transfected by Lipofectamine2000 for COS7 and 293T cells and LipofectamineLTX for SHSY5Y cells.

### Plasmids, Antibodies and siRNAs

NAT1 R64W and WT were cloned into p3XFLAG-CMV7.1 (Sigma) vectors and expressed as FLAG-tagged proteins^22^. Myc-Parkin R42P plasmid was provided by Dr. Ted Dawson (The Johns Hopkins University School of Medicine) and untagged Parkin R42P and Parkin fused at the C-terminal end to the Sec61β transmembrane region (amino acids 71–91) cloned into pCDNA3.1 vectors. BFP-Sec61β, mCherry-Sec61β and mCherry-Rab7 were kindly provided by Dr. Gia Voeltz (University of Colorado Boulder). EGFP was amplified from pIRES2-EGFP (Clontech) and fused at the N-terminus to FLAG-tagged NAT1 R64W or WT, or Parkin constructs. Plasmids for Vimentin, LC3B, Ambra1 and ubiquitin were purchased from Addgene or the DNASU plasmid repository and sub-cloned into BFP-Sec61β mCherry-Rab7 or p3XFLAG-CMV7.1 vectors.

Antibodies used in this study include anti-FLAG, anti-Parkin, anti-Calnexin, anti-TGN46, and anti-GAPDH from Sigma; anti-β-actin, and anti-LC3B from Cell Signaling; anti-Calnexin, anti-LAMP2a and anti-Rab4 from Abcam; anti-RPS6 from Santa Cruz Biotechnology.

Chemical treatment followed the table below.[Table t1]

### Live cell imaging by confocal microscopy

293T or COS7 cells were seeded onto 35 mm glass-bottom dishes 24 hours before transfection with different combinations of fluorescent protein-fused NAT1, Sec61β, Rab7, Vimentin or ubiquitin by using lipofectamine2000 (Invitrogen) according to the manufacturer’s instruction. Cells were imaged either in OPTI-MEM media (Invitrogen) with a Zeiss Cell Observer SD Spinning Disk confocal microscope equipped with a conditional chamber to control the temperature at 37 °C and 5% CO_2_ levels, 100x NA 1.40 EC Plan-Apo objective and photometrics QuantEM 512SC CCD; or with CO_2_-independent media (Invitrogen) using a Nikon Eclipse Ti inverted microscope enclosed in an environmental chamber at 37 °C and equipped with a Yokogawa spinning disc confocal and back-illuminated pixel EMCCD camera (Andor, DU897 or DU888) as well as 100x NA 1.42 Plan-Apo objective lens or 60x NA 1.45 Plan-Apo TIRF objective lens. The lasers used in all experiments were at 405 nm, 488 nm and 561 nm. Single optical sections are shown for the imaging experiments, and maximum intensity projection provided for time lapsed experiments (Fig. 4a and Supplementary Movie S1-S3).

### Super-resolution structured illumination microscopy

293T cells were cultured on #1.5 glass coverslips placed into 6-well plates and transfected with plasmids encoding fluorescent fusion proteins by using Lipofectamine 2000. Cells were washed once with PBS and immediately fixed with ice-cold 100% methanol for 7 minutes at −20 °C. Coverslips were washed with PBS 5 times for ~10 minutes and mounted with media (100 mM Tris, pH 8.5, 90% glycerol, 1 mg/mL PDDA) containing TetraSpec microspheres (Invitrogen).

SHSY5Y cells stably expressing Parkin R42P were cultured similarly and transfected with mCherry-Sec61β plasmid by using Lipofectamine LTX. Cells were washed once with PBS, fixed with 1.5% formaldehyde in PBS for 10 minutes at room temperature, and permeablized with 0.5% Triton X-100 on ice for 5 minutes. Coverslips were first blocked with 5% BSA in PBS for 30 minutes at 37 °C, incubated with mouse anti-Parkin antibody in PBS for 1 hour at 37 °C, and then goat anti-mouse IgG-Alexa 488 (Invitrogen) in PBS for 1 hour at 37 °C after washing. Nuclei were stained with 10 μg/mL DAPI on ice for 4 minutes and coverslips mounted as described above.

Super-resolution light microscopy was performed with a Nikon Structured Illumination Microscope (N-SIM), equipped with a Nikon SR Apo TIRF objective (NA 1.49) and iXon3 EMCCD camera (DU-897E, Andor Technology, Belfast, UK).

### ER enrichment by CaCl_2_ precipitation

CaCl_2_ precipitation for ER-enrichment was performed according to the procedure described in the ER isolation kit (Sigma) and as previously described^28^. 293T or SHSY5Y cells were harvested and resuspended in isotonic extraction buffer containing 10 mM HEPES pH 7.8, 250 mM sucrose, 1 mM EGTA and 50 mM potassium chloride supplemented with protease inhibitor cocktail (Roche). After 10 strokes with a Dounce homogenizer, the lysate was subjected to differential centrifugation at 1,000 g for 10 minutes and then 12,000 g for 15 minutes to generate a PMF in the supernatant. 7 volumes of 8 mM CaCl_2_ was added dropwise to the PMF during continuous stirring at 4 °C. After 15 minutes of additional stirring at 4 °C, the sample was centrifuged at 8,000 g for 10 minutes. As a control, the procedure was also performed with H_2_O addition and no CaCl_2_.

### Sucrose gradient fractionation of microsomes and Na_2_CO_3_ extraction

The sucrose gradient fractionation is adapted from a recently published protocol^30^. 293T cells were treated with 30 μg/mL cycloheximide for 5 minutes to stabilize polysomes before harvesting. 0.9 volume of 2.5 M sucrose in HM buffer (10 mM HEPES pH 7.8, 1 mM EGTA, 50 mM KCl, 0.5 mM MgCl_2_ and 1 mM DTT) was added to the PMF produced as above to adjust the sucrose concentration to 1.3 M. A step gradient was prepared from the bottom to top containing 1.5 mL of 1.8 M sucrose in HM buffer, 1.5 ml of 1.5 M sucrose in HM buffer, 5 mL of the adjusted 1.3 M sucrose-PMF mixture, 1.5 mL of 1.0 M sucrose in HM buffer, 1.5 mL of 0.6 M sucrose in HM buffer and 1.5 mL of 0.25 M sucrose in HM buffer. Ultracentrifugation was done with a SW41 swinging bucket rotor at 35,000 rpm for 19 hours at 4 °C and fractions of 700 μL were taken from the top.

Sodium carbonate extraction was done by incubating 200 μL rough microsome containing fractions from the gradient with 4 mL extraction buffer containing 200 mM Na_2_CO_3_, pH 11.5, 10 mM DTT, 0.5 M sucrose, 3% glycerol and EDTA-free protease inhibitor cocktail, on ice for 30 minutes. Samples were then centrifuged at 230,000 g at 4 °C for 1 hour. The pellet was dissolved with SDS-loading buffer and the supernatant was subjected to TCA precipitation by mixing with equal volume of 20% trichloroacetic acid and incubating on ice for 30 minutes before solubilizing with SDS loading buffer.

### Denaturing immunoprecipitation

293T cells were lysed with 7.2 M urea supplemented RIPA buffer (25 mM Tris HCl pH 7.6, 150 mM NaCl, 1% NP-40, 1% sodium deoxychloate, 0.1% SDS and protease inhibitor cocktail), briefly sonicated to reduce viscosity, incubated at 4 °C for 30 minutes, followed by centrifugation at 16,000 g for 10 minutes. The CaCl_2_ precipitated pellet fraction was dissolved in 7.2 M urea supplemented RIPA buffer directly, whereas proteins in the supernatant fractions were dissolved in 7.2 M urea supplemented RIPA buffer after TCA precipitation. All samples were diluted 8 fold with RIPA buffer before immunoprecipitation.

For immunoprecipitation, samples were precleared with protein G Sepharose beads (Sigma) for 1 hour before incubation with antibodies for 3 hours at 4 °C. Interacting proteins were then pulled down with protein G Sepharose beads with 3-hour incubation at 4 °C and eluted with SDS loading buffer after extensive washing in buffer containing 50 mM Tris HCl pH 7.5, 150 mM NaCl and 1% Triton.

### Cycloheximide chase

293T cells were transfected with plasmid DNA, as indicated in the text. 18 hours after DNA transfection, cells were treated with 30 μg/mL cycloheximide and harvested at 0, 1, 3, or 5 hours of treatment. Cells were lysed with buffer containing 50 mM Tris pH 7.5, 150 mM NaCl, 1% Triton X-100, 1 mM EDTA and protease inhibitor cocktail (Roche) for 30 minutes and centrifuged at 16,000 g for 10 minutes. Protein concentrations were measured by Pierce® 660 nm Protein Assay Reagent and 20 μg of protein used for SDS-PAGE and immunoblotting with indicated antibodies and ECL reagent (GE Lifesciences). Films were quantified by ImageJ after scanning and data plotted with Microsoft Excel. Protein abundance was normalized to β-actin.

## Additional Information

**How to cite this article**: Liu, F. *et al.* Artificial targeting of misfolded cytosolic proteins to endoplasmic reticulum as a mechanism for clearance. *Sci. Rep.* **5**, 12088; doi: 10.1038/srep12088 (2015).

## Supplementary Material

Supplementary Information

Supplementary Video 1

Supplementary Video 2

Supplementary Video 3

## Figures and Tables

**Table 1 t1:** Experimental treatment conditions.

Agent	Quantity (μM)	Duration (hours)	Cell type
MG132 (R&D systems)	2	6	293T
	10	4	COS7
	10	7	SHSY5Y
Carfilzomib (Selleckchem)	1	6	293T
Chloroquine (Sigma)	30	6	293T
	4	COS7	
Cycloheximide (Sigma)	30 μg/mL	1, 3, 5	COS7/293T/SHSY5Y
