# Supplementary material for: Artificial targeting of misfolded cytosolic proteins to endoplasmic reticulum as a mechanism for clearance
Source: Sci Rep. 2015 Jul 14;5:12088. doi: 10.1038/srep12088 (PMC4501007; doi:10.1038/srep12088)
Supplement: Supplementary Information [file srep12088-s1.pdf]

Supplementary Information

Artificial targeting of misfolded cytosolic proteins to endoplasmic reticulum as a mechanism for clearance

Fen Liu[1], Deanna M. Koepp[2], and Kylie J. Walters[1*]

[1]Protein Processing Section, Structural Biophysics Laboratory, Center for Cancer Research, National Cancer Institute, Frederick, MD 21702 USA

[2]Department of Genetics, Cell Biology and Development, University of Minnesota, Minneapolis, MN 55455 USA

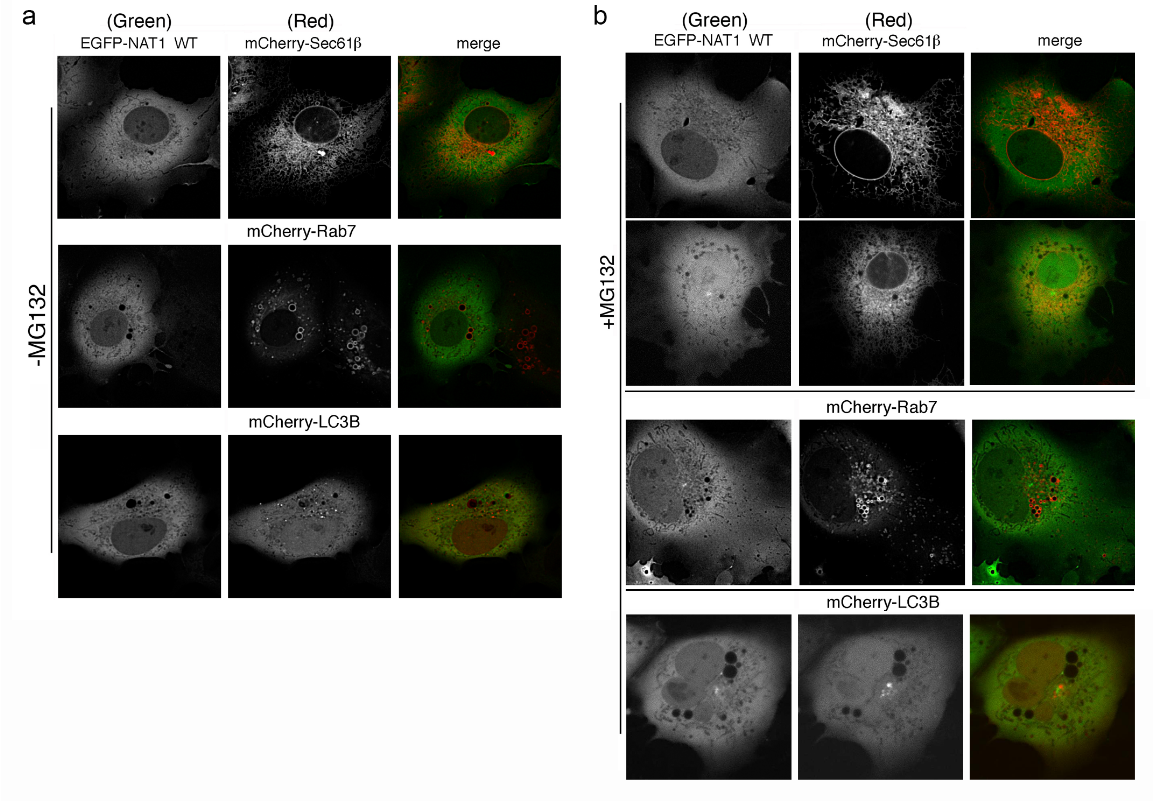

Supplementary Figure 1. NAT1 WT is a cytosolic protein that does not co-localize with Sec61β, Rab7 or LC3B. 293T cells expressing EGFP-NAT1 WT and mCherry-Sec61β (top), mCherry-Rab7 (middle), or mCherry-LC3B (bottom) without (a) or with (b) MG132 treatment were captured by live cell spinning disc confocal microscopy.

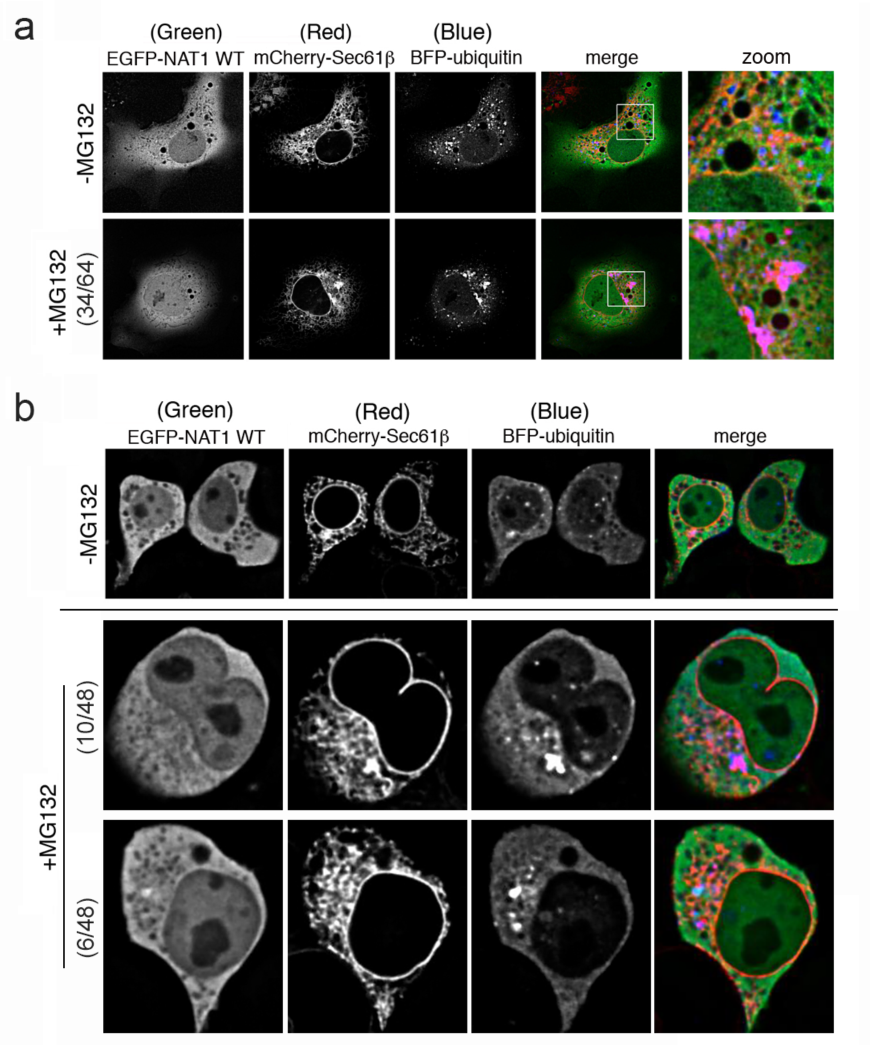

Supplementary Figure 2. NAT1 WT does not co-localize with Sec61β, but in some cells, ubiquitin co-localizes with Sec61β and NAT1 WT following MG132 treatment. COS7 (a) or 293T (b) cells expressing EGFP-NAT1 WT, mCherry-Sec61β and BFP-ubiquitin without (top) or with (bottom) MG132 treatment were captured by live cell spinning disc confocal microscopy. In (a) and the middle panel of (b), co-localization is between Sec61β and ubiquitin whereas in the bottom panel of (b), co-localization is for NAT1 WT and ubiquitin. Fractions to the left of the images indicate the number of cells showing the representative phenotype over the total number of cells examined.

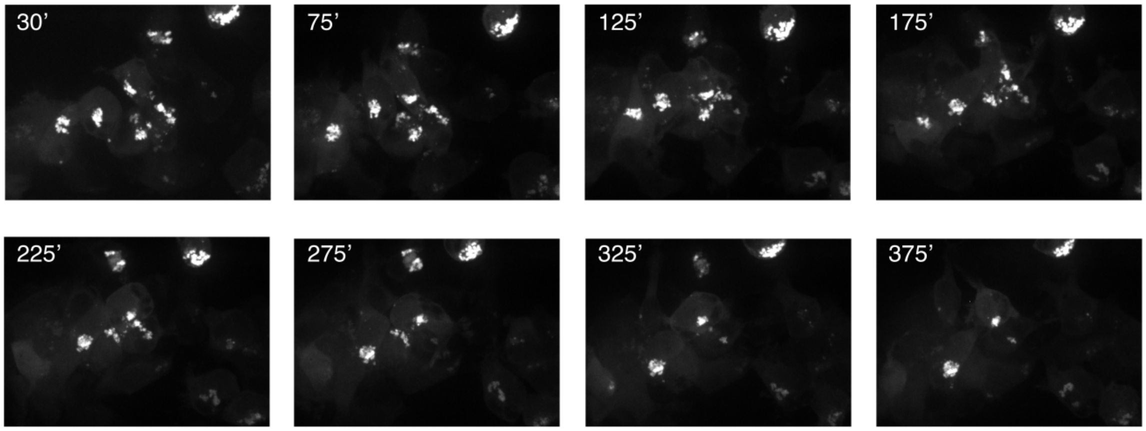

Supplementary Figure 3. NAT1 R64W is present in cells following release from proteasome inhibition when cycloheximide is absent. Time lapsed confocal microscopy experiments revealed no significant decrease of EGFP signals over time in MG132-treated 293T cells expressing EGFP-NAT1 R64W and mCherry-Sec61β. Following treatment with 2 μM MG132 for 6 hours, the culture media was exchanged to fresh media lacking MG132. Cells were imaged every five minutes by spinning disk confocal microscopy. Maximum intensity projection on EGFP channel is shown for each time point.

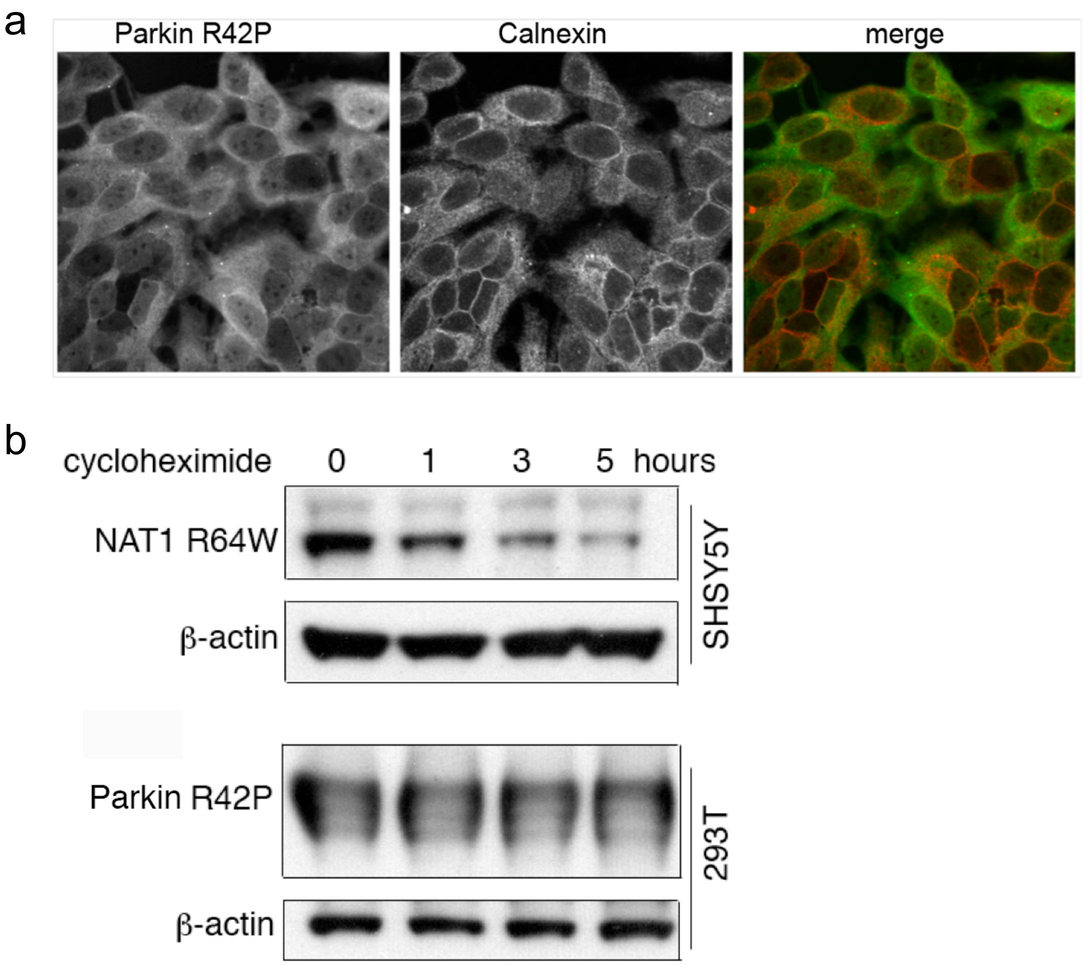

Supplementary Figure 4. Parkin R42P does not co-localize with ER marker Calnexin in SHSY5Y cells and degradation rates of Parkin R42P and NAT1 R64W are cell type independent. (a) SHSY5Y cells stably expressing Parkin R42P were fixed and indirect immunofluorescence confocal microscopy analyses done with mouse anti-Parkin (green) and rabbit anti-Calnexin (red) antibodies. (b) FLAG-NAT1 R64W and myc-Parkin R42P were expressed in SHSY5Y or 293T cells, respectively, and the cells treated with cycloheximide 18 hours later for 0, 1, 3, and 5 hours. Protein abundance was evaluated by immunoblotting cell lysates.

Supplementary Movie 1. EGFP-NAT1 R64W is cleared following release of proteasome inhibition and inhibited protein synthesis.

293T cells expressing EGFP-NAT1 R64W and mCherry-Sec61β and treated for six hours with 2 μM MG132 were exchanged into fresh media containing 30 μg/mL cycloheximide and lacking MG132. Cells were imaged in 5-minute intervals by a spinning disk confocal microscope and in this movie, maximum intensity projection of EGFP signal is displayed.

Supplementary Movie 2. A portion of EGFP-NAT1 R64W co-migrates with mCherry-Sec61β following release of proteasome inhibition and inhibited protein synthesis.

293T cells expressing EGFP-NAT1 R64W and mCherry-Sec61β and treated for six hours with 2 μM MG132 were exchanged into fresh media containing 30 μg/mL cycloheximide and lacking MG132. Cells were imaged in 5-minute intervals by a spinning disk confocal microscope and in this movie, maximum intensity projection of EGFP and mCherry are displayed.

Supplementary Movie 3. EGFP-NAT1 R64W is present in cells following MG132 release when cycloheximide is not present.

293T cells expressing EGFP-NAT1 R64W and mCherry-Sec61β and treated for six hours with 2 μM MG132 were exchanged into fresh media lacking MG132. Cells were imaged in 5-minute intervals by a spinning disk confocal microscope and in this movie, maximum intensity projection of EGFP and mCherry are displayed.
